# Complementary and alternative medicine use amongst Malaysian orthopaedic oncology patients

**DOI:** 10.1186/1472-6882-14-404

**Published:** 2014-10-17

**Authors:** Amreeta Dhanoa, Tze Lek Yong, Stephanie Jin Leng Yeap, Isaac Shi Zhung Lee, Vivek Ajit Singh

**Affiliations:** Jeffrey Cheah School of Medicine and Health Sciences, Monash University, 47500 Bandar Sunway, Malaysia; Department of Orthopaedic Surgery (Noceral), University of Malaya Medical Centre, 59100 Kuala Lumpur, Malaysia

**Keywords:** Complementary medicine, Alternative, Orthopaedic, Oncology, Cancer, Tumour, CAM

## Abstract

**Background:**

Although studies have shown that a large proportion of cancer patients use CAM, no study on CAM use amongst orthopaedic oncology patients has been published. Therefore, this study aims to determine the prevalence, characteristics and factors associated with CAM use amongst orthopaedic oncology patients.

**Methods:**

All consecutive consenting patients/parents who presented at the Orthopaedic Oncology Clinic, University Malaya Medical Centre (1st January to 31st December 2013) were interviewed using a structured questionnaire.

**Results:**

Overall, one hundred sixty-eight of the 274 patients recruited (61.3%) had used CAM at some time during their current illness. The prevalence of CAM used was 68% (123/181) for patients with malignant tumours and 48.4% (45/93) for patients with benign tumours. The most popular CAMs were biological-based therapies (90.5%), followed by mind-body techniques (40.5%). The most frequently used biological therapies were mega/multivitamins (31%), snakehead (*Chana striatus*) (28%) and sea cucumber (*Stichopus horrens*) (18%); whereas prayers (31%) and holy water (13%) dominated the mind-body category. Common reasons for CAM use were to improve physical well-being (60.1%), try out everything that would help (59.5%) and to enhance wound-healing (39.3%). Independent predictors for CAM use in multivariate analysis were paediatric patients [OR 2.46; 95% CI 0.99–6.06; p = 0.05], malignant tumours [OR 1.90; 95% CI 1.12–3.25; p = 0.018] and patients who underwent surgery [OR 2.06; 95% CI 1.15–3.69; p = 0.015]. Majority patients started taking CAMs following suggestions from family members (53%) and friends (49%). Sixty-six percent of patients felt they actually benefitted from CAM and 83.3% were satisfied/very satisfied. Only 5 patients reported side-effects. Majority of CAM users planned to continue CAM use or recommend it to others. However, only 31.5% of patients disclosed their CAM usage to their doctors.

**Conclusions:**

This survey revealed a high prevalence of CAM usage amongst orthopaedic oncology patients, with majority patients expressing satisfaction towards CAM. Oncologists should proactively ask patients about CAM to prevent potential adverse effects, as most patients do not share this information with them.

## Background

The desire among human beings to explore beyond the realms of conventional medical treatment is clearly illustrated by the usage of complementary and alternative medicine (CAM) during illness [1, 2]. This usage is more pronounced when patients have difficult to treat chronic medical ailments such as chronic pain, poor mental health and cancer for which conventional medicine may not provide effective and satisfactory remedies [1, 2]. Cancer ranks amongst the most dreaded of all illness [3]. As the diagnosis, symptoms and treatment of cancer challenges every dimension of a person’s life including physical, emotional, mental and spiritual aspects [3], patients with cancer are more receptive to CAM utilization. CAM are defined as medical and healthcare systems, practices, and products that are not currently considered an integral part of conventional medicine [4].

Many CAM users report use not so much as a result of being dissatisfied with conventional medicine, but largely because they found these healthcare alternatives to be more congruent with their own values, beliefs, and philosophical orientations toward health and life [1]. Although the effectiveness and safety of certain CAM are questionable, studies have reported widespread CAM usage amongst cancer patients to increase body’s ability to fight cancer, improve quality of life, strengthen immune system and cope with disease symptoms [1, 5–7]. Others use CAM as a last resort and as a way of finding hope [3, 7, 8]. Essentially, CAM meets the demands that mainstream medicine cannot principally satisfy, and provides complementary solutions to conventional medicine [9].

The increasing popularity of CAM amongst cancer patients is well recognized. A recent European survey reported that the overall prevalence of CAM used by cancer patients was 35.9%, ranging from 14.8% to 73.1% [5]. There has been a noticeable increase in CAM usage amongst cancer patients, from 25% in the 1970–1980s to 32% in the 1990s and 49% after 2000 [10]. As cancer incidence increases and survival time lengthens, patients using CAM are likely to escalate [7]. This trend is gradually recognized by oncologists, although not many patients discuss these therapies with them [7].

Evidence-based recommendations regarding the usefulness and safety or lack thereof of CAM practices in oncology setting continues to evolve [11–17]. An increasing number of evidence-based studies support the use of CAM therapies as a complement to conventional cancer therapies. For instance, Chinese medicinal herbs have been shown to reduce treatment side-effects, improve quality of life and survival rates amongst adult cancer patients [11]. Hypnosis lessens pain, anxiety and distress during procedures and appears to be especially effective amongst paediatric cancer patients [12]. Medical Qigong has been shown to improve quality of life, mood and fatigue and reduces inflammation amongst cancer patients [13]. Despite the growing scientific evidence favoring CAM usage, numerous reports highlighting the adverse-effects and risks to patient safety resulting from direct effect or possible interactions with conventional therapies are emerging [14]. St John’s wort reduces plasma levels of the active metabolites of three chemotherapy agents: irinotecan, imatinib mesylate, and docetaxel, with potential deleterious consequences on treatment outcome [15]. Although kava-containing products were found to be effective in controlling anxiety, stress and insomnia, recent reports associate this herbal remedy with severe hepatotoxicity [16]. Garlic, ginkgo, and ginseng, have been associated with postoperative bleeding via platelet inhibitory effects [17].

To date, numerous studies highlighting the growing use of CAM amongst cancer patients have been reported. These studies report either on patients with wide arrays of cancers or focuses on specific cancer particularly breast, prostate, gynecological and colorectal cancers [8]. However, to our best knowledge, this study is the first to specifically explore CAM usage amongst patients with orthopaedic oncology tumours. Another important distinction regarding this study is that it involves patients of all age groups and encompasses both malignant and benign tumours. This is unlike most other studies that involve either adults or children and focus solely on malignant tumours [6–8, 10, 18–20]. Advances in treatment in paediatric oncology field, have resulted in an increase of 5-year survival rate of patients to approximately 80% [21]. One important consequence of this paradigm shift is that survivors of childhood cancer are likely to encounter acute and long-term adverse-effects resulting from the illness or mainstream medical therapies [22]. This drives many parents and patients to use CAM to manage disease symptoms, treatment side-effects and repeated painful operations despite the paucity of data supporting their efficacy and safety [12, 22].

Thus, an exhaustive coverage is integral towards understanding CAM usage amongst orthopaedic oncology patients, since this field comprises both malignant and benign tumours and involves all age-groups. Therefore, this study was conducted amongst patients with orthopaedic oncology tumours to determine: the prevalence, characteristics, predictors and reasons for CAM use, types of CAM therapies and practices used, satisfaction with CAM, source of information and disclosure to doctors.

## Methods

### Study design, setting and study population

This was a prospective, cross-sectional study conducted from 1st January to 31st December, 2013 at the Orthopaedic Oncology Clinic, University Malaya Medical Centre (UMMC), a 1051-bedded tertiary referral centre situated in Kuala Lumpur. As the leading referral centre for orthopaedic oncology patients in Malaysia, its patient population is reflective of a larger community in Malaysia. The study was approved by the Medical Ethics Committee of UMMC (Reference No: 944.12).

Patients with histologically confirmed bone and soft tissue tumours, diagnosed for at least 2 months were recruited consecutively as they presented to the clinic. Informed consent was obtained from patients or parents/caregivers for patients < 18 years. Patients were excluded if they were unable to give informed consent or if their condition precluded the ability to do so.

### Questionnaire

For questionnaire development, an extensive literature search exploring CAM usage amongst cancer patients was conducted. Pre-test of the questionnaire was undertaken to obtain feedback from 6 orthopaedic oncology patients. Patients were asked to suggest CAMs not already in the existing list. The pre-test feedback and information captured from healthcare professionals, was used to develop a culturally relevant and comprehensive questionnaire. Four interviewers were trained to administer the questionnaires. To increase reliability and validity of responses, face-to-face interviews were conducted using structured questionnaire.

The questionnaire comprised three main sections. The first section captured basic socio-demographic data, such as age, gender, religion, marital status, highest education level and income. The second section captured disease-related characteristics such as tumour type, presence of metastasis, time since diagnosis and treatment. This information was cross-checked with medical notes. Bone and soft tissue tumours were classified according to World Health Organization (WHO) classification [23].

Each patient was presented with a list of CAMs on the questionnaire. These CAMs were broadly divided into 5 modalities based on National Centre for Complementary and Alternative Medicine (NCCAM, 2005) and Cochrane CAM Field categorization [24, 25]. These were: biological, spiritual/mind-body, alternative, physical (body-based) and energy therapies. Patients were then asked if they had used any of the listed CAMs or any other CAMs not listed, either before or after their diagnosis. Patients were considered CAM users if they had used CAM at least once and non-users if they had not used any CAM during the current illness. Patients who had been using CAM even before their diagnosis were regarded non-users, unless a change in pattern of usage was demonstrated after diagnosis, e.g. increased dose or frequency. The third section of the questionnaire captured information on types of CAMs, reasons for use, benefits attained, satisfaction, source of information and disclosures to doctors. For paediatric patients, the questionnaire was answered mainly by their parents. Non-users were acknowledged after completing the socio-demographic and clinical section and their interview ended here.

Data was analysed using SPSS version 20.0, comparing CAM users and non-users. Categorical variables were compared using Fisher’s exact test or Chi-squared test (*p* < 0.05). Variables associated with CAM usage in the univariate analysis were entered into multivariate logistic regression analysis. Where appropriate, CAM use was compared in patients with malignant verses benign tumours and in adult verses paediatric patients.

## Results

### Prevalence, clinical characteristics and socio-demographic status

During the one year study period, 285 patients were interviewed, with complete data obtained from 274 patients. There were 181 (66.1%) patients who presented with malignant tumours and 93 (33.9%) with benign tumours. The various bone and soft tissue tumours (WHO, 2013) [23] are presented in Table 1. Mean age was 41.27 ± 20.53 years (range 8 to 90 years).

**Table 1:** **Histological diagnosis (WHO Classification, 2013)** [23] **(n = 274)**

Malignant tumours of Bone and Soft Tissue (N = 181)		
Bone (n = 109)		
	Osteogenic tumours^a^	37
	Metastasis to bone	33
	Miscellaneous tumors^b^	17
	Chondrogenic tumours^c^	13
	Haematopoietic neoplasms	8
	Tumours of undefined neoplastic nature	1
Soft tissue (n = 72)		
	Undifferentiated/unclassified sarcoma	22
	Adipocytic tumours	17
	Tumors of uncertain differentiation	16
	Fibroblastic/myofibroblastic tumors	8
	Nerve sheath tumor	4
	Smooth muscle tumors	3
	Vascular tumors of soft tissue	1
	Skeletal muscle tumors	1
**Benign tumours of Bone and Soft Tissue (n = 93)**		
Bone (n = 55)		
	Osteoclastic giant cell rich tumors	36
	Tumor of undefined neoplastic nature	10
	Chondrogenic tumor	8
	Osteogenic tumor	1
Soft tissue (n = 38)		
	Adipocytic tumor	13
	Vascular tumor of soft tissue	9
	Fibroblastic/Myofibroblastic tumor	5
	Nerve sheath tumor	4
	Fibrohistocytic tumor	3
	Benign tumor of uncertain differentiation	4

^a^All were osteosarcoma, ^b^64.7% were Ewing sarcoma, ^c^All were chondrosarcoma.

Overall, 168 patients (61.3%) had used CAM at some time during their current illness. The prevalence of CAM used was 68% (123/181) for patients with malignant tumours and 48.4% (45/93) for patients with benign tumours. Overall, 75.2% patients underwent surgery, 26.6% underwent chemotherapy and 18.6% radiotherapy. Majority patients used CAM either concurrently with conventional therapy (44%) or upon completion of conventional treatment (44.6%); whereas 11.3% started CAM before conventional therapy but stopped upon commencing conventional treatment.

### Predictors for using CAM

Factors associated with CAM usage in univariate analyses (p < 0.05) (Table 2) were paediatric patients, malignant tumours, metastasis, patients who underwent chemotherapy and surgery. Multivariate analysis revealed that paediatric patients (OR 2.46; 95% CI 0.99–6.06; p = 0.05), malignant tumours (OR 1.90; 95% CI 1.12–3.25; p = 0.018) and surgery (OR 2.06; 95% CI 1.15–3.69; p = 0.015) remained significant predictors. CAM use was not affected by gender, ethnicity, marital status, income, educational level and employment status.

**Table 2:** **Predictors of CAM use**

Variable	All patients (N = 274) (%)		Users of CAM N (%)	Nonusers of CAM N (%)	P	OR	95% C1
Paediatric	Yes	32 (11.7)	25 (78.1)	7 (21.9)	0.04	2.47	1.03–5.94
	No	242 (88.3)	143 (59.1)	99 (40.9)			
Age range	<20	58 (21.2)	38 (65.5)	20 (34.5)	0.43		
	21–40	77 (28.1)	43 (55.8)	34 (44.2)			
	41–60	78 (28.5)	52 (66.7)	26 (33.8)			
	>60	61 (22.3)	35 (57.4)	26 (42.6)			
Gender	Male	129 (47.1)	73 (56.6)	56 (43.4)	0.13	1.46	0.89–2.38
	Female	145 (52.9)	95 (65.5)	50 (34.5)			
Race	Malay	118 (43.1)	76 (64.4)	42 (35.6)	0.64		
	Chinese	109 (39.8)	65 (59.6)	44 (40.4)			
	Indians	47 (17.2)	27 (57.4)	20 (42.6)			
Marital status*	Married	163 (67.4)	101 (62.0)	62 (38.0)	0.19	1.44	0.83–2.47
	Not married	79 (32.6)	42 (53.2)	37 (46.8)			
Education*	Primary/secondary	167 (69.0)	94 (56.3)	73 (43.7)	0.19	1.46	0.83–2.58
	College/university	75 (31.0)	49 (65.3)	26 (34.7)			
Job status*	Employed	109 (45.0)	61 (56.0)	48 (44.0)	0.37	1.27	0.76–2.12
	Unemployed	133 (55.0)	82 (61.7)	51 (38.3)			
Income*	Less than 1000	151 (62.4)	91 (60.3)	60 (39.7)	0.63	1.14	0.67–1.93
	More than 1000	91 (37.6)	52 (57.1)	39 (42.9)			
Presence of metastasis	Yes	88 (32.1)	62 (70.5)	26 (29.5)	0.03	1.80	1.05–3.10
	No	186 (67.9)	106 (57.0)	80 (43.0)			
Malignancy	Malignant	181 (66.1)	123 (68.0)	58 (32.0)	0.002	2.26	1.36–3.78
	Benign	93 (33.9)	45 (48.4)	48 (51.6)			
Chemotherapy	Yes	73 (26.6)	55 (75.3)	18 (24.7)	0.004	2.38	1.31–4.34
	No	201 (73.4)	113 (56.2)	88 (43.8)			
Surgery	Yes	206 (75.2)	137 (66.5)	69 (33.5)	0.002	2.37	1.36–4.14
	No	68 (24.8)	31 (45.6)	37 (54.4)			
Radiotheraphy	Yes	51 (18.6)	35 (68.6)	16 (31.4)	0.24	1.48	0.77–2.83
	No	233 (81.4)	133 (59.6)	90 (40.4)			

N = 274 for all variables, except variables labelled * (N = 242) as variable* excludes paediatric patients.

### Reasons for using CAM

Overall, the most common reasons that drove patients to utilize CAM were to enhance overall health and physical well-being (60.1%), to try out everything that would help (59.5%) and to enhance wound healing (39.3%) (Table 3). When analysed separately, patients with malignant tumours cited the first two reasons more commonly whereas enhancement of wound healing was more common amongst patients with benign tumours. Other reasons did not differ significantly between the two groups and included controlling disease progression (32.1%), boosting immune system (27.4%) and alleviating disease symptoms and toxic effects of conventional therapy (26.2%).

**Table 3:** **Reasons for CAM use amongst 168 CAM users**

Reasons for using CAM	All patients (N = 168) (%)	Malignant (N = 123) (%)	Benign (N = 45) (%)	P ^1^	Adults (N = 143) (%)	Paediatric (N = 25) (%)	P ^2^
Enhance overall health/physical well-being	101 (60.1)	80 (65.0)	21 (46.7)	0.03	87 (60.8)	14 (56.0)	0.648
Trying everything that can help	100 (59.5)	79 (64.2)	21 (46.7)	0.04	85 (59.4)	15 (60.0)	0.958
Enhance wound healing	66 (39.3)	42 (34.1)	24 (53.3)	0.02	58 (40.6)	8 (32.0)	0.419
Control disease progression	54 (32.1)	39 (31.7)	15 (33.3)	0.84	44 (30.8)	10 (40.0)	0.362
Boost immune system to fight disease	46 (27.4)	36 (29.3)	10 (22.2)	0.36	37 (25.9)	9 (36.0)	0.295
Alleviate disease symptoms/toxic effect of conventional therapy	44 (26.2)	29 (23.6)	15 (33.3)	0.2	36 (25.2)	8 (32.0)	0.474
Improve psychological well-being and finding hope	30 (17.9)	21 (17.1)	9 (20.0)	0.66	27 (18.9)	3 (12.0)	0.574
More compatible with own value/belief towards life and health	26 (15.5)	22 (17.9)	4 (8.9)	0.15	24 (16.8)	2 (8.0)	0.374
Allow to relax and helps in sleep	24 (14.3)	20 (16.3)	4 (8.9)	0.23	20 (14.0)	4 (16.0)	0.76
Seeking a cure	23 (13.7)	17 (13.8)	6 (13.3)	0.94	19 (13.3)	4 (16.0)	0.753
Complementary effect to conventional medicine	21 (12.5)	15 (12.2)	6 (13.3)	0.84	16 (11.2)	5 (20.0)	0.207
More control over own treatment	15 (8.9)	11 (8.9)	4 (8.9)	1	12 (8.4)	3 (12.0)	0.471
Conventional treatment is too toxic	7 (4.2)	6 (4.9)	1 (2.2)	0.68	6 (4.2)	1 (4.0)	1
Dissatisfied with conventional medicine	4 (2.4)	3 (2.4)	1 (2.2)	1	4 (2.8)	0	1

Columns do not sum to 100% due to the option of multiple answers.

P^1^comparison of patients with malignant verses benign tumours.

P^2^comparison of adult verses paediatric patients.

We then compared the paediatric and adult oncology patients to determine the CAM motivators, and noted no significant differences (Table 3). Most parents indicated that they wanted to do everything possible for their child (60%) and to improve the child’s general-health (56%), paralleling the main reasons cited amongst the adult patients. However, reasons like controlling disease progression, seeking a cure, boosting immune system, alleviating side-effective of conventional therapy were notably higher amongst the paediatric patients, although not statistically significant. It was reassuring to note that only 4 patients utilized CAM because they felt dissatisfied with conventional therapy.

### Types of CAM used

The most common CAM modalities used were biological-based therapies (90.5%), followed by mind-body techniques (40.5%) (Table 4). Patients with malignant tumours were more likely to use biological-based therapies (p = 0.04). Conversely, patients with benign tumours were more likely to use physical-based therapies (0.002). Majority patients (61.5%) had used 2 or more CAM modalities. When paediatric and adult patients were compared (Table 4), biological therapies were used with equal frequencies and were the most commonly used CAMs amongst both the groups. In contrast, alternative therapy usage was significantly higher amongst the adults patients (p = 0.027). Although mind-body and physical practices were notably higher in adult patients, the difference was not statistically significant.

**Table 4:** **CAM modalities used amongst 168 CAM users**

Modalities	All patients (N = 168) (%)	Malignant (N = 123) (%)	Benign (N = 45) (%)	P ^1^	Adults (N = 143) (%)	Paediatric (N = 25) (%)	P ^2^
Biological	152 (90.5)	115 (93.5)	37 (82.2)	0.04	130 (90.9)	22 (88.0)	0.710
Mind-body	68 (40.5)	47 (38.2)	21 (46.7)	0.32	61 (42.7)	7 (28.0)	0.168
Physical	36 (21.4)	19 (15.4)	17 (37.8)	0.002	33 (23.1)	3 (12.0)	0.213
Alternative	24 (14.3)	18 (14.6)	6 (13.3)	0.83	24 (16.8)	0 (0)	0.027
Energy	15 (8.9)	12 (9.8)	3 (6.7)	0.76	13 (9.1)	2 (8.0)	1

Columns do not sum to 100% due to the option of multiple answers.

P^1^comparison of patients with malignant verses benign tumours.

P^2^comparison of adult verses paediatric patients.

The various CAMs used within each modality are detailed in Table 5. Most frequently used biological therapies were mega/multivitamins (31%), snakehead (*Chana striatus*) (28%), sea cucumber (*Stichopus horrens*) (18%), herbal remedies (13%), fish oil (13%), fruits juices with antioxidant properties (10%), spirulina (9%), and sabah snake grass (*Clinacanthus nutans*) (7.7%). Mega/multivitamins were used in combination with other CAMs in all but one patient.

**Table 5:** **Types of CAM used amongst 168 CAM users**

CAM	N	%
***Biological therapy***		
**Vitamin and mineral supplements**		
Mega/multivitamins	52	31
Calcium suplements	12	7
Antioxidant pills	7	4.2
**Herbal**		
Sea cucumber/gamat (*Stichopus horrens*)	30	18
Herbal remedies	22	13
Fruit juice with antioxidant properties	17	10
Spirulina (*Arthrospira plantensis*)	15	9
Belalai gajah/sabah snake grass (*Clinacanthus nutans*)	13	7.7
Other medicinal plant	9	5.4
Medicinal mushroom supplements	9	5.4
Aloe vera	8	4.8
Apricot seed	7	4.2
Ginseng	7	4.2
Evening primrose	6	3.6
Green tea	6	3.6
Habatus sauda seeds	6	3.6
Medicinal tea	5	3
Ginko biloba	5	3
**Non-herbal**		
Snakehead/haruan (*Chana striatus*)	47	28
Fish oil	22	13
Porcupine quill powder/dates	8	4.8
Honey	6	3.6
Special water	5	3
Birds nest	5	3
**Special diet**	10	6
***Physical therapy***		
Massage therapy	33	19.6
Reflexology	2	1.2
Chiropractic	1	0.6
Cupping	2	1.2
***Mind-body medicine***		
Prayer/faith healing	52	31
Holy water/zam zam water	22	13
Traditional healers	9	5.4
Meditation	8	4.8
Yoga	4	2.4
Support group	4	2.4
Mental imagery	1	0.6
***Energy therapy***		
Bioelectromagnetic therapy	7	4.2
Qi Gong	3	1.8
Manual healing	2	1.2
Taichi	2	1.2
Reiki	1	0.6
Ionic foot bath	1	0.6
Longevitology	1	0.6
***Alternative therapy***		
Acupuncture	16	9.5
Traditional Chinese medicine	10	6
Traditional Indian medicine	2	1.2
Homeopathy	2	1.2

Please note that some respondents reported use of more than one type of CAM within a CAM category; thus, response values in this table will not necessarily add up to the values presented in Table 4. Additionally, response totals are thus greater than total participants.

The most common mind-body techniques were prayers (31%) and holy water (13%), whereas, various message techniques (19.6%) dominated the physical therapy category. After excluding prayer healing, the overall CAM prevalence remained almost the same (60%), as almost all patients used other CAM modalities alongside prayer healing. Other popular CAM methods were acupuncture (9.5%), traditional healers (5.4%) and various traditional Chinese medicines (6%). The most common CAMs used amongst the 206 patients who underwent surgery were snakehead and multivitamins/megavitamins (21.36% each respectively) followed by sea cucumber (9.7%).

Amongst the paediatric oncology patients, multi/megavitamins were the most frequently used CAM (56%). Other biological-based therapies commonly used amongst children were spirulina (20%), freshwater eel (16%), and antioxidant pills (16%). Mind-body medicine (28%) was the second most common CAM modality used; (prayer/faith healing, n = 5, holy water, n = 5 and traditional healer, n = 1). On the contrary, various physical and energy therapies seemed unpopular amongst paediatric oncology patients; only 4 patients used them (massage, n = 2, cupping, n = 1, bioelectromagnetic therapy, n = 1). Conspicuously, none of the alternative therapies were used by the paediatric oncology patients; all 16 patients who used acupuncture were adult patients.

### Satisfaction with CAM

Overall, majority of CAM users were very satisfied/satisfied (83.3%) with no significant differences in CAM satisfaction rates between malignant and benign tumour group (p = 0.1) In response to an open-ended question, 66% of CAM users felt they actually experienced positive effects which were feeling more energetic and stronger (45%), enhanced wound healing (22.5%), pain relief (18%), calmer mind and better sleep (11.7%), tumour shrinkage (5.4%), improved appetite (5.4%), fewer side-effects from chemotherapy (3.6%), internal cleansing and body detoxification (3.6%) and suppression of disease progression (2.7%). Majority of CAM users (67%) planned to continue CAM use or recommend it to others. Similarly, majority parents (84%) were satisfied with CAM use and 64% felt that their child had actually benefitted in some way.

Only 5 patients (4 adults and 1 child) experienced unwanted effects which were; enlargement of swelling after traditional message (n = 2), facial swelling after consuming Chinese medication (n = 1), heatiness after consuming mushroom- based capsules (n = 1) and excessive coughing after consuming sabah snake grass (n = 1). Only 3 patients discontinue conventional medicine for CAM; the reasons cited were cost, side-effects and fear of radiotherapy.

### Source of information and disclosure

When asked how CAM modalities were selected, majority indicated family members (53%) and friends (49%) as their main source, followed by mass media/websites (24%) and own-will (18.5%). Only 14.3% of CAM usage was based on recommendations by healthcare personnel (Table 6). When analysed separately, the only difference noted was that patients with benign tumour were more likely to start CAM resulting from their own initiative. Majority (68.5%) of CAM users did not discuss these therapies with their doctors. The non-disclosure rates were similar for patients with malignant verses benign tumors (p = 0. 941) and for adult verses paediatric patients (p = 1). The 3 most common reasons for non-disclosure were; ‘felt it was unnecessary’ (42.6%), ‘doctors did not ask’ (35%) and ‘afraid to tell as doctor may not agree’ (10.6%). For patients who did disclose, the doctors responded as follows; remained neutral (51%), encouraged (35.8%) and discouraged (13.2%) CAM use. Only 25.6% of patients were proactively asked about their CAM usage by their doctors.

**Table 6:** **Source of information amongst 168 CAM users**

Source	Total (N = 168) (%)	Malignant (N = 123) (%)	Benign (N = 45) (%)	p
From family members	89 (53.0)	61 (49.6)	28 (62.2)	0.15
From friends	82 (49.0)	63 (51.6)	19 (42.2)	0.28
From mass media/websites	40 (24.0)	28 (23.0)	12 (26.7)	0.62
Your own free will	31 (18.5)	16 (13.0)	15 (33.3)	0.003
From health personnel	24 (14.3)	18 (14.6)	6 (13.3)	0.83
From other patients	20 (12.0)	15 (12.3)	5 (11.1)	0.83
From CAM practitioner	16 (9.5)	13 (10.6)	3 (6.7)	0.56
From your church/religious group	5 (3.0)	3 (2.4)	2 (4.4)	0.61

Columns do not sum to 100% due to the option of multiple answers.

## Discussion

To our best knowledge, this study is the first to specifically explore CAM usage amongst patients with orthopaedic oncology tumours. Since there have been no other similar studies, we related our findings to other comparable studies, depending on the context of discussion. The prevalence of CAM used amongst orthopaedic oncology patients was high. Overall, 61.3% patients used at least one CAM modality during their current illness. CAM usage was significantly higher amongst patients with malignant tumours compared with their benign tumours counterparts (68% versus 48.4%, p = 0.002); concurring with a nationwide survey amongst Japanese cancer patients which displayed higher CAM usage in malignant tumours compared with benign tumours; albeit at lower rates (45% versus 26%) [26].The rate of 68% noted amongst patients with malignant tumours was higher than the prevalence reported from previous studies amongst cancer patients [6, 10, 18, 26–28]. In a large European survey [5], the highest prevalence of CAM was in pancreatic (56.3%), liver (55.6%), bone/spinal (54.5%) and brain cancer (50%) patients. However, the number of bone/spinal cancer patients was small (n = 22), representing 2.3% of patients in this series.

Apart from malignant tumours, the other independent predictors for CAM use were paediatric patients and patients who underwent surgery. A considerable proportion of paediatric patients used CAM, with significantly higher rates than adults (78% versus 59%, p = 0.04). Likewise, high CAM usage (84.5%) was noted in another study involving Malaysian children with cancer [20]. In a systematic review, CAM prevalence amongst children with cancers ranged from 6% to 91% [19]. This has been linked to lower survival perspective and fewer days since relapse [29], suggesting that CAM therapies may act as coping strategies for families in efforts to try everything possible to cure or alleviate the pain and distress in their children. Although we did not determine the effects of parenteral socio-demographic and education characteristics on CAM practices in children, no such influence was found in another study involving Malaysia children with cancer [20]. Children whose parents used CAM were more likely to use CAM compared to children whose parents did not [30]; this is not unexpected since children are dependent on their parents for their healthcare decisions.

Concurring with another study [7], patients who underwent surgery for tumour removal were more likely to use CAM, particularly various biological modalities. This finding should not be underestimated, as clinical complications during perioperative period can result from natural therapies, either from direct effect or herb-drug interaction [17]. Thus, patients scheduled for surgery should be questioned about none prescription medications usage [17]. In retrospect, we should have added a question regarding CAM timing in relation to the surgery.

Patients with metastasis and those who received chemotherapy were more likely to use CAM, although these factors were not predictive in multivariate analysis. While some studies reveal higher CAM usage amongst patients with metastatic cancers [18], others suggest no association with advanced disease [7, 20]. Stage of disease impacted patients’ reasons and expectations for CAM usage, although it may not predict CAM use [7]. Although higher education and socio-economic status have been linked to CAM usage [1, 5, 8, 18]; concurring with other studies we found no such association [7]. Reasons that catalyze CAM use in countries with higher socio-economic status, might work in opposite directions in lower socio-economic status countries [8]; the latter attributed to cultural factors and more traditional lifestyle.

It is important for doctors to recognize the reasons motivating patients’ CAM usage. Our patients were mainly motivated by the desire to try out everything possible to fight the disease, to improve their general health and to enhance wound healing. The first two reasons emerged at significantly higher rates in patients with malignant compared with benign tumours, suggesting that these patients may be turning to CAMs as additional measures to enhance general overall health, rather than focusing solely on their illness; supporting the philosophical congruence theory [1]. CAM use was also fuelled by the desire to control disease progression, boost the immune system and to cope with treatment and/or its side-effects. Reassuringly, only 4 patients attributed dissatisfaction with conventional medicine as a reason for using CAM. Our findings indicate that patients are not deserting conventional therapies which have been thoroughly tested, for unconventional treatment which lack scientific evidence regarding safety and efficacy.

Due to the physical and psychological impacts of an oncology condition, coping strategies are essential tools for patients and parents to move through the illness trajectory. Both biological therapies and mind-body techniques were most frequently used amongst our patients and its use may be explained by Lazarus and Folkmans’ problem-focused and emotion-focused coping strategies [31, 32]. Problem-focused strategies include the use of various biological therapies in attempts to alleviate disease-related symptoms or adverse-effects of conventional treatment, while prayers and meditation techniques are examples of emotional-focused coping strategies as patients venture into a spiritual and emotional journey [18]. Factors that drive patients and parents towards the uptake of CAM may also be viewed from a problem-focused verses emotion –focused perspective. Overall, when the factors motivating CAM usage were analysed, they were skewed towards problem-focused factors (>20% frequency each); these included enhancing physical well-being, accelerating wound-healing, controlling disease progression, boosting immune system and ameliorating symptoms resulting from disease and treatment. Parents of paediatric oncology patients also seemed lop-sided to-wards problem-focused factors. During conventional treatment, parents often play a passive role with little opportunity for active involvement in the healing process of their children [33]. Coping strategies focusing on problem-solving are action-centred and enables more involvement in care activities of their child [33]. Other reasons such as improving psychological well-being and finding hope, taking more control over own treatment, allowing to relax/sleep may be described as emotion-focused factors and were less frequently mentioned (<20% frequency) in our study.

Majority of our patients were satisfied with CAM usage and would continue its use and even recommend to others. We believe that the satisfaction shown was because the expectations for CAM were met. Majority of our patients took CAM with the hope of enhancing their physical well-being and healing process, whilst only a small proportion took CAM with the intention to cure their illness. This mirrors the reasons for taking CAM and the resulting satisfaction amongst Singaporean cancer patients [27]. Conversely, disappointment towards CAM was high amongst Nigerian cancer patients, as majority used CAM to treat or cure cancer, with unmet expectation ultimately [34].

Biological-based therapies were the most common CAM used, with vitamins, snakehead and sea cucumber, constituting the three most common biological therapies. Vitamins were widely used in multiple series [5, 7, 27], reflecting self-promoting behaviour contributing to general health. A substantial proportion of our patients embarked on various prayer healing methods and almost all patients used these techniques alongside other CAM modalities. Prayers and spiritual therapies are important aspects amongst Malaysian oncology patients [35] and strong connections between reliance on religious beliefs and the ability to cope with cancer has been shown [3], contributing to psychosocial adjustment to cancer and its treatment.

While the variances of what constitutes CAM possibly contribute to the diverse prevalence rates reported in literature, we believe the multi-ethnicity of our patients with its diverse cultural background, religious beliefs and practices contribute to the high prevalence and differential patterns of CAM use. Worldwide these practices differ depending on cultural habits, geographic locations, ethnic backgrounds and religious beliefs. Majority of Japanese cancer patients used products such as mushrooms, herbs and shark cartilage [26], whereas spiritual practices and vitamins which were widespread in our study and Western countries [7] were rarely used amongst the Japanese cancer population [26]. Singapore cancer patients commonly used traditional Chinese medicine, bird’s nest and special diets [27], whereas in Turkey, herbal therapies such as stinging nettle were used widely [28]. While spiritual approaches, vitamins and herbs were the most common CAMs used in USA [7], Australian cancer patients used mainly dietary supplements, prayers, herbs and relaxation techniques/meditation [18]. Generally, natural products are widely used amongst cancer patients emphasizing an appeal for ‘natural’ remedies, as many patients believe natural equates to safe.

While energy, alternative and mind-body medicine had similar usage across patients with benign and malignant tumor, biological therapies were more common amongst patients with malignant tumors. Contrariwise, physical therapies involving various massage therapies were more common amongst patients with benign tumor. Although massage therapies alleviate anxiety and distress symptoms amongst cancer patients, rare complications such as hae-morrhage and fractures can occur [36], especially with pre-existing weakened bone from underlying pathologies. Acupuncture used by 10% of our patients, has been shown to relieve cancer related pain and chemotherapy induced nausea and vomiting [37]. However, it should be used cautiously in patients with deranged clotting mechanisms e.g. with impaired liver function, as bleeding can occur [37]. Only 2.4% of our patients used support groups, suggesting a lack of support group amongst patients with musculoskeletal tumours.

Children are more likely to use biologically-based therapies and mind-body therapies compared with alternative or energy therapies [30], concurring with the findings of our study. In a study conducted amongst children and adolescents with cancer, adolescents showed a tendency towards using more invasive therapies (e.g., acupuncture) compared to children [38].

Majority of the patients seemed to detach CAM and conventional medicine by not disclosing CAM use to their doctors, with most patients having the perception that disclosure was unnecessary. Disclosure rates of 20% to 77% have been reported [39] and the main reasons for non-disclosure were doctor’s lack of inquiry; patient’s anticipation of the doctor’s disapproval, disinterest, or inability to help and patient’s perception that CAM disclosure is irrelevant to conventional care [39].

Our study supports the role of family members and friends on patient’s CAM uptake. Significant others (SOs) such as family members, partners and close friends influence decisions about CAM use amongst cancer patients, often acting as information providers and active decision makers [40, 41]. Qualitative studies also indicate the importance of a supportive social network and the involvement of SOs in uptake and maintenance of CAM, contributing to bonding between patients and their SOs [41].

Many patients relied on information from unscientific sources, such as mass media and internet. However, these patients may be at risk of adverse effects as many websites promote CAM without scientifically proven safety and efficacy data [42]. Only 14.3% of our patients received information regarding CAM from healthcare professionals. This may be due to the fact that most healthcare professionals feel they lack adequate knowledge or were not up-to-date with the best evidence on CAM use in oncology [43].

Few limitations of this study must be acknowledged. The drawback of face-to-face compared to anonymous questionnaire is that participants may not disclose certain information for fear of negative repercussions. However, face-to-face interview allows respondents and interviewers to seek clarification, increasing the response rates and reliability of answers. Secondly, although we did attempt to provide an exhaustive list of CAMs to ensure consistency in defining CAMs and to reduce recall bias; participants might still have been less likely to report the non-listed CAMs. Although this study was confined to a single institution, being one of the main orthopaedic oncology referral centre in this country, it receives majority of referrals in this region. Thus, this study could be taken as a proxy indicator for CAM usage amongst orthopaedic oncology patients.

## Conclusions

This study highlights a high prevalence of CAM usage amongst orthopaedic oncology patients, especially amongst patients with malignant tumors, paediatric patients and patients who undergo surgery. Patients appear to use CAM in conjunction with, not instead of conventional medicine. Biological-based therapies were the most popular CAM therapies used. Majority patients used CAM with the hope of enhancing their physical well-being and healing process and were generally satisfied with the CAMs. Given the high prevalence of CAM usage amongst orthopaedic oncology patients, oncologists should proactively ask their patients about CAM usage as most patients do not voluntarily disclose this information. In order to foster better patient-doctor communication, oncologists should be familiar with the commonly used CAM amongst patients, or at least be able to direct them to reliable sources of information.
